# Titanium Nitride as a New Prospective Material for NanoSQUIDs and Superconducting Nanobridge Electronics

**DOI:** 10.3390/nano11020466

**Published:** 2021-02-12

**Authors:** Michael I. Faley, Yuchen Liu, Rafal E. Dunin-Borkowski

**Affiliations:** 1 Peter Grünberg Institute 5, Forschungszentrum Jülich GmbH, 52425 Jülich, Germany; yuchen.liu@rwth-aachen.de (Y.L.); r.dunin-borkowski@fz-juelich.de (R.E.D.-B.); 2 Faculty of Georesources and Materials Engineering, RWTH Aachen University, 52062 Aachen, Germany; 3 Ernst Ruska-Centre for Microscopy and Spectroscopy with Electrons, Forschungszentrum Jülich GmbH, 52425 Jülich, Germany

**Keywords:** titanium nitride, nanobridge Josephson junction, nanoSQUID, superconducting electronics

## Abstract

Nanobridge Josephson junctions and nanometer-scale superconducting quantum interference devices (nanoSQUIDs) based on titanium nitride (TiN) thin films are described. The TiN films have a room temperature resistivity of ~15 µΩ·cm, a superconducting transition temperature *T_c_* of up to 5.3 K and a coherence length *ξ*(4.2 K) of ~105 nm. They were deposited using pulsed DC magnetron sputtering from a stoichiometric TiN target onto Si (100) substrates that were heated to 800 °C. Electron beam lithography and highly selective reactive ion etching were used to fabricate nanoSQUIDs with 20-nm-wide nanobridge Josephson junctions of variable thickness. X-ray and high-resolution electron microscopy studies were performed. Non-hysteretic *I*(*V*) characteristics of the nanobridges and nanoSQUIDs, as well as peak-to-peak modulations of up to 17 µV in the *V*(*B*) characteristics of the nanoSQUIDs, were measured at 4.2 K. The technology offers prospects for superconducting electronics based on nanobridge Josephson junctions operating within the framework of the Ginzburg–Landau theory at 4.2 K.

## 1. Introduction

Superconducting electronics exhibit lower energy consumption, higher speed and lower noise than semiconductor electronics. However, the miniaturization of superconducting circuits is challenging. A reduction in the thickness and width of superconducting lines to below ~100 nm can lead to an increase in their kinetic inductance. Smaller Josephson junctions (JJs) without shunting resistances and higher critical current densities *J_c_* are required. High values of *J_c_* are needed to ensure a sufficiently large Josephson energy *E_J_* for stability against thermal fluctuations, as well as to squeeze magnetic flux bias into nanometer-sized loops of superconducting quantum interference devices (SQUIDs) or cells of rapid single flux quantum (RSFQ) circuits. The use of nanobridge JJs is advantageous for the miniaturization of SQUIDs and other superconducting circuits because they provide the highest possible values of *J_c_*, which can approach the depairing critical current density, in superconducting films (~30 MA/cm^2^ in Nb thin films).

Nanobridge JJs have negligible capacitance when compared with tunnel JJs. However, they often have hysteretic *I*(*V*) characteristics due to overheating or a multivalued current-phase dependence *I*(*φ*). Thermal hysteresis is observed in nanobridge JJs that have sufficiently large critical currents *I_c_*, while the *I*(*φ*) dependence becomes multivalued when the length *L* of a nanobridge JJ exceeds the coherence length *ξ* by a factor of ~3.5 [1], depending on the shape of the nanobridge. In Nb thin films, *ξ*(0 K) is ~10 nm, resulting in the need for sophisticated 10 nm preparation technology for non-hysteretic operation [2,3,4]. Nb-based nanobridge JJs that have a length of ~100 nm show non-hysteretic operation only over a narrow temperature range above 7.2 K [5]. The absence of hysteresis in their *I*(*V*) characteristics at higher temperature results from a reduction in *I_c_* of the JJ in the case of thermal hysteresis and from an increase in *ξ* as the temperature *T* of the nanobridge approaches the superconducting transition temperature *T_c_*, according to the relation ξ∝(1−TTc)−12.

It would be more practical to operate nanobridge JJs in hysteresis-free mode at a temperature of 4.2 K, which is naturally stabilized by the boiling of liquid He at atmospheric pressure. A nanobridge length of ~100 nm can be achieved relatively easily and with adequate reproducibility by using reactive ion etching (RIE). However, Nb nanobridges of this size are hysteretic at 4.2 K as a result of their short coherence length. Instead of Nb, a superconducting material that has a value of *T_c_* of ~5 K and a value of *ξ* in a thin film of ~50 nm would be more suitable. Here, we suggest using titanium nitride (TiN) for this purpose, despite the fact that nanobridge JJs were not realized previously from TiN films.

Covalent bonding between Ti and N is responsible for the hardness and chemical stability of TiN. It is therefore used to harden surfaces and to protect them against corrosion, as well as for decorative coatings that resemble Au [6]. TiN can be classified as a quantum material as a result of its superconducting and plasmonic properties. Coplanar waveguide resonators based on superconducting TiN films have demonstrated internal quality factors of up to 10^7^ [7]. Resonators based on TiN films have much fewer losses than resonators made with Nb, Re or Al films deposited on the same substrates [8]. The use of superconducting TiN films in shunting capacitors in transmon qubits was found to enhance relaxation and dephasing times six-fold over those obtained using lift-off Al up to ~60 µs [9]. In the latter study, the JJs in the transmon qubits were still Al tunnel JJs made using shadow evaporation, which may have been one of the residual sources of decoherence. It would be important to realize all-TiN qubits to minimize dielectric losses arising from dissipation due to quantum two-level states (TLSs) at oxide interfaces [10], especially in the oxide that forms at the metal–air interface [8].

Epitaxial 8 × 8 μm^2^ TiN/AlN/TiN tunnel JJs were fabricated on MgO and characterized [11]. TiN and MgO have lattice constants of approximately 0.42 nm and similar rocksalt crystal structures, simplifying epitaxial growth. However, a low critical current density *J_c_* of ~50 A/cm^2^ and a large subgap leakage current were observed. These features are disadvantageous for applications of tunnel TiN JJs in nanoscale superconducting circuits. The deposition of TiN films on Si substrates would be preferable to minimize the density of TLSs and the oxygen content of TiN, as well as for technological compatibility with other processes, including bulk nanomachining of Si substrates for the fabrication of cantilevers [4]. However, Si has a diamond crystal structure with a lattice constant of 0.54 nm, which corresponds to a 28% lattice misfit with TiN, complicating epitaxial growth of TiN films on Si. Nevertheless, four-unit cells of TiN match three-unit cells of Si with less than 4% misfit, making it possible to realize domain matching epitaxy [12].

In this paper, we report the fabrication and characterization of the first TiN nanobridge JJs, as well as nanoscale SQUIDs (nanoSQUIDs) based on these JJs. We observed that the superconducting transition temperature of TiN films grown on Si was higher than that of TiN films grown under the same conditions on MgO or sapphire. In combination with other practical advantages, such as availability and price, ease of nanomachining and low noise, this determined our choice to study TiN films and devices prepared on Si substrates. The large coherence length *ξ* of 100 nm at 4.2 K in the TiN films allows relatively easy patterning of variable thickness nanobridge JJs that have length ~100 nm, *J_c_* ~ 10 MA/cm^2^ and non-hysteretic *I*(*V*) characteristics at 4.2 K. NanoSQUIDs based on variable thickness nanobridge JJs have relatively low kinetic inductance of their loops and up to ~17 µV peak-to-peak modulations of their *V*(*B*) characteristics at 4.2 K. Furthermore, the corrosion resistance of TiN provides long-term stability of nanoSQUIDs and other superconducting circuits based on TiN nanobridge JJs.

## 2. Materials and Methods

TiN films were deposited by pulsed reactive DC magnetron sputtering from 50 mm TiN targets, primarily on 10 mm × 10 mm × 0.4 mm Si (001) substrates. The edges of the substrates were oriented along the (110) and (1$\bar{1}$0) crystallographic planes of Si. MgO and sapphire substrates were also tested for comparison. The substrates were cleaned in an ultrasonic bath successively in acetone, propanol and double-distillated deionized water. The native oxide was removed from the Si substrate by rinsing in a 10% HF solution for 20 s, followed by cleaning in double-distillated deionized water and drying by blowing with 99.9995% pure N_2_. The substrates were placed on a heater plate in the sputtering system, which was pumped to a base pressure of 4 × 10^−8^ mbar. The TiN films were sputtered at a rate of ~1 nm/min in a gas mixture Ar(80%)-N_2_(20%) at a total pressure of 10^−2^ mbar, a DC voltage bias of 200 V, a DC current of 100 mA and a heater temperature of 920 °C, which corresponded to a substrate temperature *T_sub_* of ~800 °C. Before deposition, the chamber was outgassed at the deposition conditions for more than 1 h without plasma and for 10 min of pre-sputtering on the shutter in its “CLOSED” position between the target and the substrate. The gases that were used for sputtering TiN were of the best available purity: 99.9999% pure Ar and 99.9999% pure N_2_, each containing below 0.01 ppm of oxygen, corresponding to a partial pressure of oxygen in the sputtering plasma of below 10^−10^ mbar.

The microstructural properties and crystallographic orientations of the TiN films were studied using high-resolution scanning electron microscopy (HRSEM), high-resolution transmission electron microscopy (HRTEM) and X-ray diffraction (XRD) in Forschungszentrum Jülich (FZJ) (Jülich, Germany). HRTEM images were recorded using a Tecnai F20 TEM at an accelerating voltage of 200 kV. 2Θ XRD scan patterns were acquired using a BRUKER D8 DISCOVER high resolution X-ray diffractometer with an in-plane rotation angle of *φ* = 45°. The dependences of the resistance of the TiN films on temperature *R*(*T*) and magnetic field *R*(*B*) were measured using a Quantum Design Physical Property Measurement System (PPMS). Voltage modulations of the nanoSQUIDs with magnetic field *V*(*B*) at different current biases and temperatures were measured using the same PPMS. The *R*(*T*) characteristics of the nanobridges and *I*(*V,B*) characteristics of nanoSQUIDs were measured using a Quantum Design DynaCool system in FZJ. The dependence of the induced magnetic moment of the superconducting TiN films on magnetic field was measured using a Quantum Design Magnetic Property Measurement System (MPMS) at 1.9 K, which is the lowest stabilized temperature in this system.

Patterning of the TiN films with a spatial resolution down to 10 nm was performed by RIE using masks of hydrogen silsesquioxane (HSQ) electron beam resist. The ~60-nm-thick 3% HSQ resist was exposed using an electron beam with an accelerating voltage of 100 kV, a current of 10 nA and a dose of 2.5 mC/cm^2^. The design of the nanoSQUID included a third electrode, which was used for direct injection of flux bias [2,4]. Submicrometer structures were written in the resist using a 2-nm-diameter electron beam, while wider structures of current lines were written using a 10 nm electron beam. Contact pads were etched through Pt thin film masks that were deposited through a Ti shadow mask. A post-exposure bake was performed for 2 min at 120 °C. The resist was developed in 25% tetramethylammonium hydroxide (TMAH) for 20 s in an ultrasonic bath, before rinsing the sample in distillated and deionized water in an ultrasonic bath for 1.5 min following by rinsing in deionized water for 1 min. RIE of the TiN films was performed in the clean room of the Helmholtz Nano Facility (HNF) in FZJ [13] using an Oxford Instruments PlasmalabSystem 100 with pure SF_6_ gas and only 25 W of radio frequency (RF) plasma, which provided a selectivity of ~10 for etching the TiN films, as compared to the etch rate of the HSQ resist. Inductively coupled plasma was not used during RIE because this would reduce selectivity.

More than 100 TiN thin film samples were produced and investigated during the last two years of optimization of the deposition parameters and nanostructuring technology. Approximately half of them were produced at the current deposition conditions and only their dimensions and the shapes of the nanobridges were adjusted. The superconducting transition temperatures of TiN films of similar thicknesses had a spread that was within 1%. The spread of critical currents of the nanobridges was correlated with the spread in their dimensions of approximately 20% due to the sensitivity of the reactive ion etching rate to the surface cleanliness of the TiN films and the preconditioning of the RIE machine.

## 3. Results

**Figure 1:**
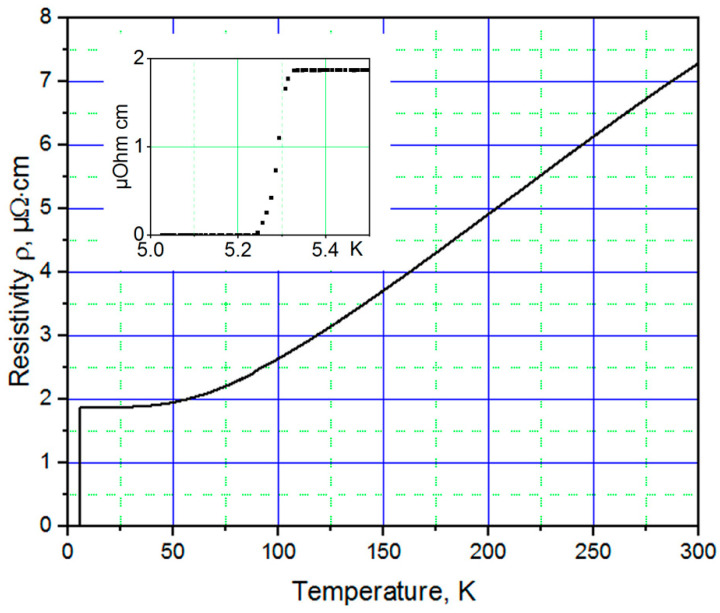
*R*(*T*) dependence of a 700-nm-thick titanium nitride (TiN) film grown on a Si (100) substrate. The inset shows *R*(*T*) dependence at the superconducting transition.

The lowest room temperature resistivity of ~15 µΩ·cm, the largest residual resistance ratio RRR = R_300K_/R_10K_ ≅ 4 and the largest superconducting transition temperature *T_c_* ≅ 5.25 K were measured for 700-nm-thick TiN films grown on Si substrates (see Figure 1). A similar room temperature resistivity was measured in [12] for epitaxial TiN films whose stoichiometry was confirmed using Rutherford backscattering spectrometry.

This value of *T_c_* is higher than that of TiN films grown under the same conditions on MgO (4.65 K) or sapphire (3.7 K). Moreover, the value of *T_c_* for the TiN films was lower when using pristine Si substrates with native oxide on their surfaces (4.8 K) that had not been removed using 10% HF during substrate preparation. TiN films that were deposited on Si covered by a 20-nm-thick SiN buffer layer demonstrated sufficiently high values of *T_c_* ≅ 5.1 K.

The superconducting transition temperature *T_c_* of the TiN films depends strongly on deposition conditions and film thickness. The value of *T_c_* is higher when using a lower base pressure and a higher substrate temperature *T_sub_*. A reduction in film thickness to below ~150 nm leads to an exponentially decreasing *T_c_*: 100-nm-thick TiN films deposited on oxide-free Si (100) substrates have *T_c_* ≅ 4.86 K and a residual resistance ratio RRR of ~2.

**Figure 2:**
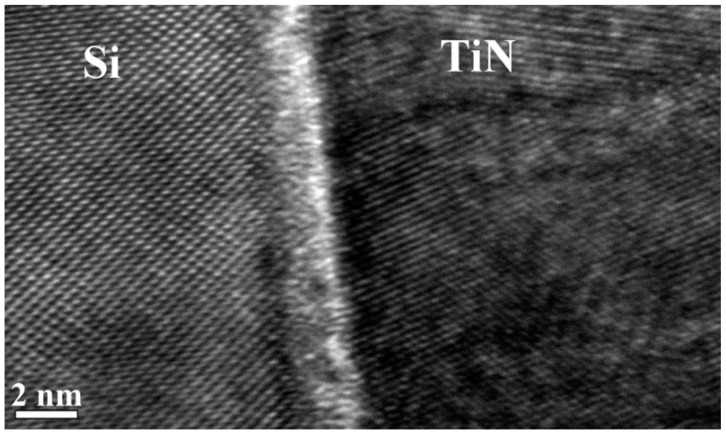
High-resolution TEM image of a Si (110) cross-section, showing (100) planes in the TiN film and (111) planes Si substrate, with the interface between them oriented parallel to the electron beam.

Figure 2 shows a cross-sectional HRTEM image of the interface between a Si substrate and a TiN film viewed along the [110] crystallographic direction of Si. The angle between (100) planes in the TiN film and the surface of the Si (100) substrate of approximately 70° is consistent with a (111) orientation of the TiN film. On a larger scale, the TiN film shows a polycrystalline structure with a typical crystallite size of 5–50 nm. The crystallite size increases with film thickness.

The (100) planes of the (111) oriented TiN film cross the (100)-oriented surface of the Si substrate. Four unit cells along the (100) planes of the TiN film (4 × 0.424 nm) match three unit cells of the (100)-oriented Si substrate (3 × 0.543nm) with a 4% misfit. This can provide a weak coupling between the TiN film and Si surface. Similar misfits have been reported for epitaxial (200)-oriented TiN films that were prepared using pulsed laser deposition [12].

**Figure 3:**
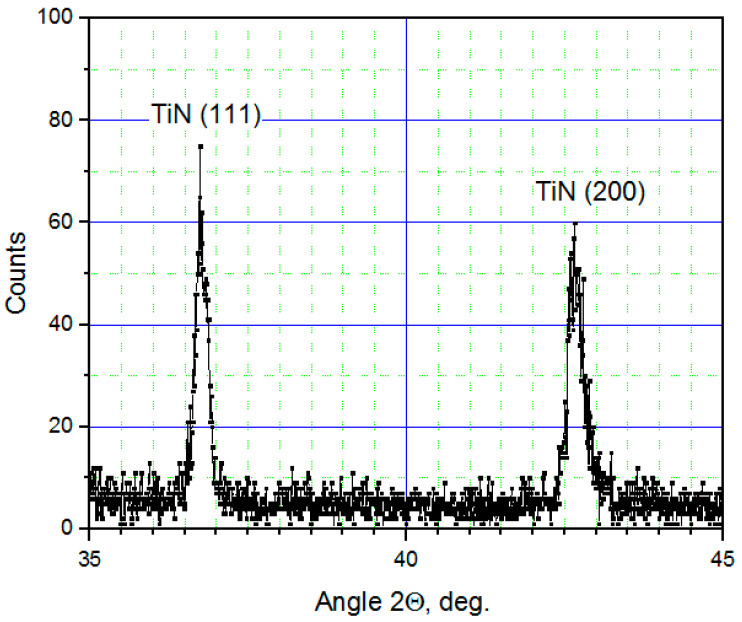
XRD scan recorded from a 400-nm-thick TiN film deposited on an oxide-free Si substrate.

The (111) orientation is predominant for TiN films that have a thickness of below 200 nm, while thicker films also contain a (200) orientation contribution. Figure 3 shows an XRD scan recorded from a 400-nm-thick TiN film grown on an oxide-free Si substrate, containing peaks from both (111) and (200) orientations. A smaller ratio between the amplitudes of the (200) and (111) peaks and a smaller value of *T_c_* were observed for thinner TiN films and/or lower deposition temperatures *T_sub_*. The amplitude of the (200) TiN peak increased relative to the (111) peak with increasing film thickness.

**Figure 4:**
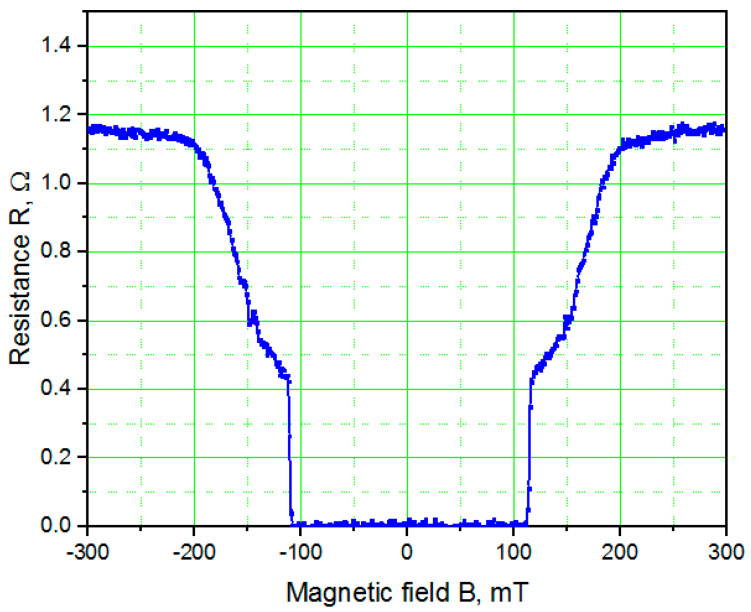
Magnetoresistance *R*(*B*) of a 100-nm-thick TiN film recorded at 1.8 K. The magnetic field was oriented perpendicular to the surface of the film.

Figure 4 shows the magnetoresistance *R*(*B*) of a 100-nm-thick TiN film recorded using PPMS with the magnetic field applied perpendicular to the film surface at 1.8 K. The upper critical magnetic field B_c2_ is approximately 0.21 T, which corresponds to a superconducting coherence length *ξ*(1.8 K) of $\sqrt{\Phi_{0}/2\pi B_{C2}}\;$≅ 39 nm. This value is significantly larger than the value of *ξ*_Nb_ of ~10 nm measured for Nb thin films, which is beneficial for the fabrication of JJs. Moreover, at 4.2 K the coherence length increased to ~107 nm as a result of the temperature dependence ξ∝(1−TTc)−12. The irreversibility magnetic field *B_irr_* ≅ 0.12 T corresponds to the threshold magnetic field for creep of Abrikosov vortices, which is therefore the upper limit of the magnetic field for low noise operation of Josephson devices.

**Figure 5:**
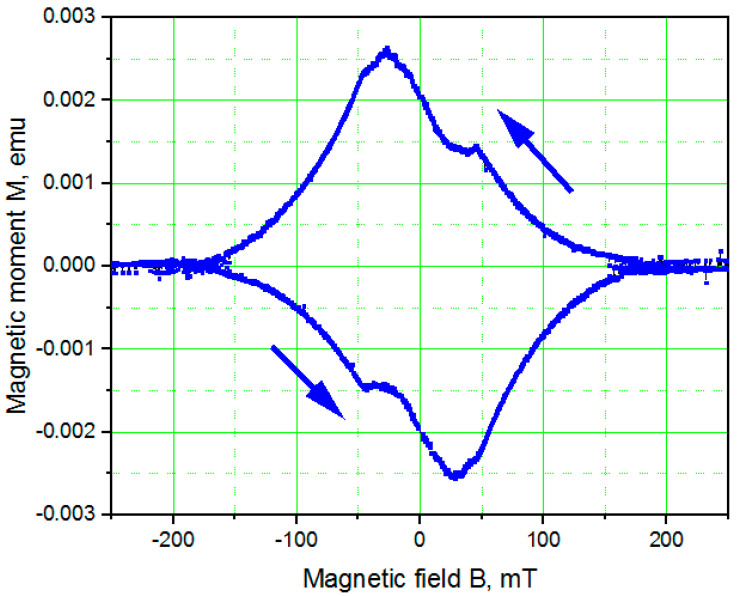
Dependence of magnetic moment *M*(*B*) of a 1.2-µm-thick TiN film deposited on a Si substrate with a 20-nm-thick SiN buffer layer recorded at 1.9 K. The magnetic field was applied parallel to the surface of the film.

Figure 5 shows the dependence of magnetic moment on magnetic field *M*(*B*) for a 1.2-µm-thick TiN film that had been deposited on a Si substrate with a 20-nm-thick SiN buffer layer measured using MPMS at 1.9 K. The magnetic field was applied parallel to the film surface. For such a thicker TiN film, the irreversibility magnetic field *B_irr_* of ~50 mT is lower, as a result of weaker pinning of Abrikosov vortices in films that have better crystallinity. The upper critical magnetic field *B_c2_* of ~0.15 T corresponds to a coherence length *ξ*(1.9 K) of ~50 nm. At 4.2 K, the coherence length increased as a result of the temperature dependence ξ∝(1−TTc)−12 to ~105 nm, which is approximately the same as that measured for thinner films because the larger *ξ*(1.9 K) is compensated at 4.2 K by the higher value of *T_c_*.

**Figure 6:**
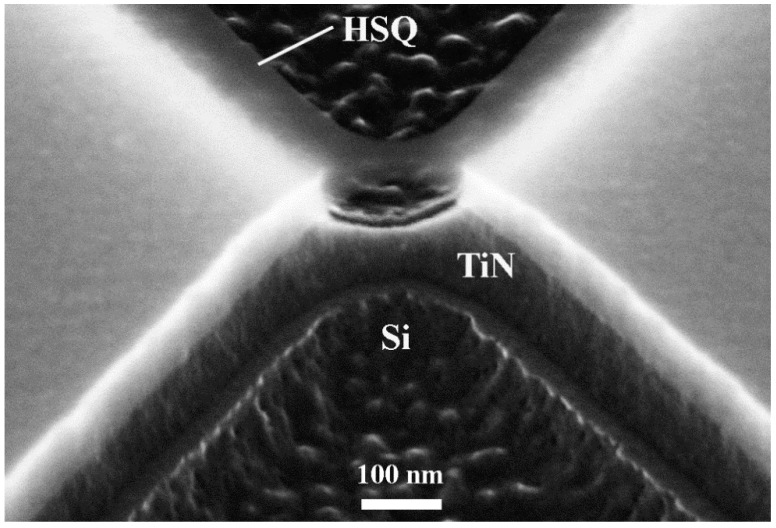
High-resolution SEM image of a TiN nanobridge Josephson junction (JJ) recorded at a 45° sample tilt angle.

Nanobridge JJs were fabricated from 100-nm-thick TiN films using 30-nm-wide bow-tie-type bridges in the mask of the HSQ resist. During RIE, isotropic etching with a 50 nm undercut was observed, as shown in Figure 6.

As a result of the undercut, the JJ takes the form of a variable thickness nanobridge that is much thinner and narrower in its middle part. The cross-section is a triangle, with an average width of ~20 nm that is much smaller than the coherence length in the TiN film *ξ*(4.2 K) ≅ 105 nm. After overcoming a small threshold, likely due to the presence of a thin SiN layer that grew naturally during presputtering at the interface between Si and TiN, the RIE continued into the Si substrate.

**Figure 7:**
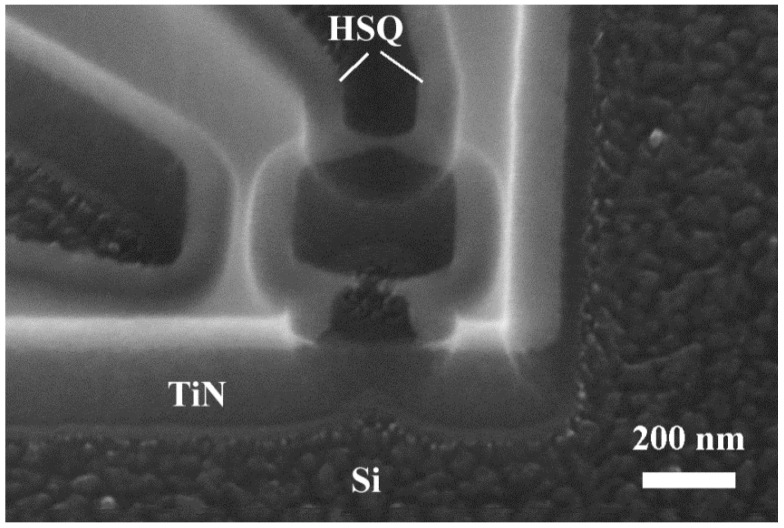
High-resolution SEM image of a TiN nanometer-scale superconducting quantum interference device (nanoSQUID) recorded at a 45° sample tilt angle.

Figure 7 shows an HRSEM image of a TiN nanoSQUID. The second superconducting current lead of one of the electrodes can be used for the direct injection of magnetic flux bias into the SQUID loop [2,4].

**Figure 8:**
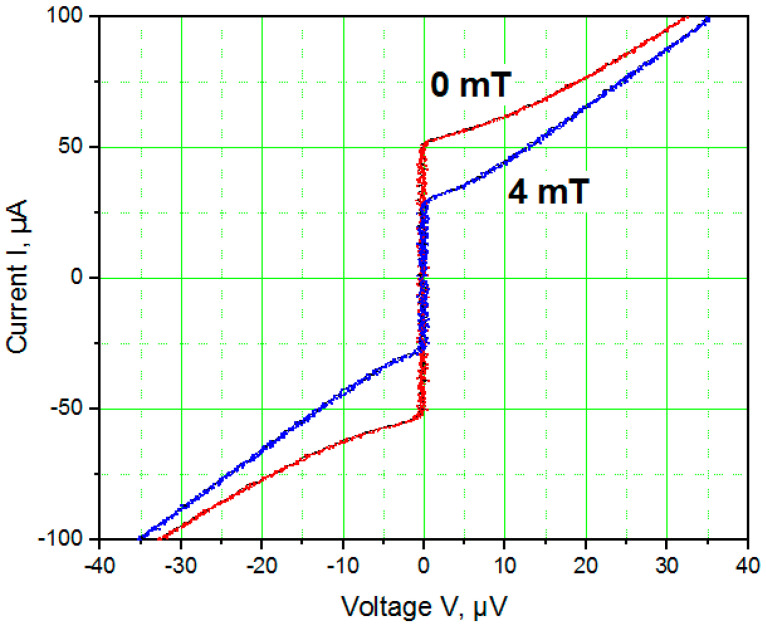
*I*(*V*) characteristics of a TiN nanoSQUID measured in magnetic fields of 0 mT and 4 mT at 4.2 K.

Figure 8 shows the *I*(*V*) characteristics of a TiN nanoSQUID measured in magnetic fields of 0 and 4 mT at 4.2 K. At zero magnetic field, this nanoSQUID has a non-hysteretic *I*(*V*) characteristic at *I_c_* ≅ 50 µA. NanoSQUIDs with thicker JJs have larger critical currents, but their *I*(*V*) curves remain non-hysteretic up to *I_c_* > 100 µA, which is much larger than the maximal critical current of a non-hysteretic Nb nanoSQUID based on nanobridge JJs [14]. The observed large amplitude of modulation is related to the relatively low kinetic inductance of a nanoSQUID with variable thickness nanobridge JJs.

**Figure 9:**
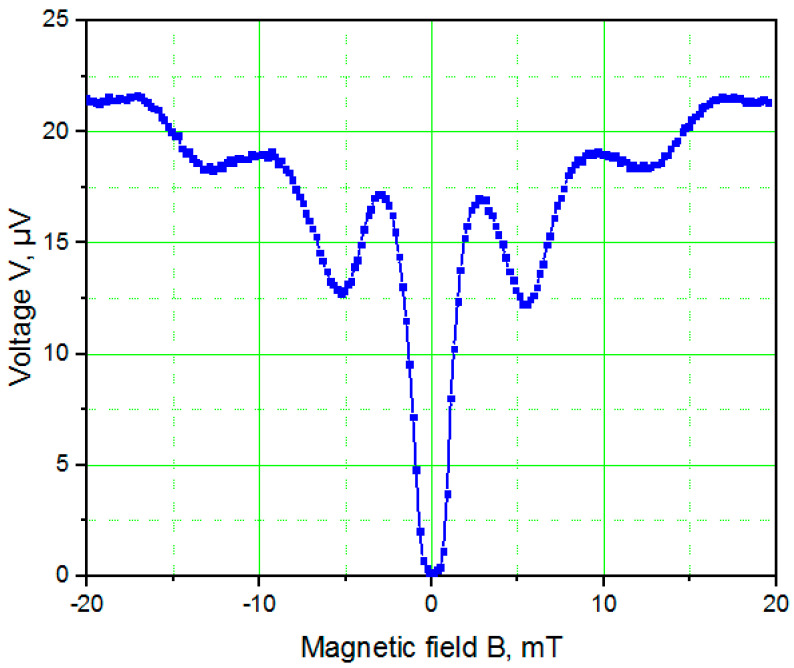
*V*(*B*) dependence of the TiN nanoSQUID measured at 4.2 K.

Figure 9 shows the *V*(*B*) dependence of the TiN nanoSQUID measured for a bias current of 60 µA at 4.2 K. For this design of nanoSQUID, the effective area and period of critical current modulation on the magnetic field are 0.36 µm^2^ and 6 mT, respectively, in accordance with earlier observations for Nb nanoSQUIDs [2,4]. The *V*(*B*) dependence of the TiN nanoSQUID is not typical for SQUIDs and looks more like the Fraunhofer diffraction pattern that is typical for the magnetic field dependence of the critical current of a single Josephson junction. This can be a consequence of the three-angle cross-sectional shapes of the nanobridge JJs. The effective area of the nanoSQUID is not exactly defined and there is an interference of different passes for Cooper pairs, which can mimic the diffraction pattern of a slit in optics (Fraunhofer pattern) and in an individual JJ.

## 4. Discussion

This is the first time that TiN has been used for the preparation of nanobridge JJs and SQUIDs. It has several properties that are advantageous for superconducting nanobridge electronics: (a) a large coherence length in thin films; (b) a superconducting transition temperature *T_c_* of ~5 K, which is appropriate for nanobridge operation at 4.2 K; (c) technological compatibility with Si; (d) long-term stability due to superior oxidation resistance and thermal and chemical stability; (e) mechanical hardness and stability; (f) low microwave losses; (g) availability and relatively low cost; (h) highly selective RIE; (i) it is a plasmonic material with tunable optical properties; (j) it has other properties that are advantageous for applications in the microelectronics industry [15].

The superconducting transition temperature *T_c_* of the TiN films depends strongly on the choice of substrate material, the deposition conditions and the film thickness. Surprisingly, TiN films have the highest values of *T_c_* when they are grown on pure Si (100) substrates, despite the 28% lattice misfit and polycrystalline microstructure of the TiN films on these substrates. This behavior may result from the minimal diffusion of oxygen from the substrate into the TiN film for a pure Si substrate. The unintended incorporation of oxygen in a TiN film is the strongest factor in suppressing superconducting properties in TiN films [16]. The use of a sufficiently low base pressure of <10^−7^ mbar in the deposition chamber and outgassing of the chamber at the deposition temperatures helps to reduce deleterious oxygen outgassing during film deposition. A substrate temperature *T_sub_* of ~800 °C was found to be optimal to achieve the highest value of *T_c_*, as well as a golden color of the 100-nm-thick TiN films.

The TiN films are polycrystalline with two preferred orientations: (111) and (200). HRTEM images show primarily a (111) orientation of the film near its interface with the Si substrate. XRD also shows that TiN films that are thinner than ~200 nm have a predominantly (111) orientation. XRD scans of thicker films show peaks corresponding to a (200) orientation. Films with (111) orientations have the lowest internal stress energy, while the (200) plane has the lowest surface energy [17,18]. The polycrystalline microstructure of the TiN films may result from the accommodation of internal stress at the interface with the substrate as a consequence of the 28% lattice misfit. An increase in film thickness reduces the contribution of interface stress energy far from the interface relative to the surface energy, leading to an increase in the proportion of (200)-oriented grains.

TiN films have the largest coherence length *ξ*(4.2 K) of ~105 nm when compared to films of other materials that are superconducting at 4.2 K. As a result of the large coherence length, the effect of grain boundaries on the critical current of a TiN film is much smaller than in Nb thin films (*ξ*(4.2 K) ≅ 15 nm) or in YBa_2_Cu_3_O_7−x_ (*ξ*(4.2 K) ≅ 1.2 nm). The polycrystalline microstructure of TiN films does not degrade their superconducting properties significantly but is beneficial for isotropic RIE during the preparation of JJs.

As a result of the large coherence length in TiN thin films, it was found that the preparation of nanobridge JJs that are non-hysteretic at 4.2 K was much easier than for nanobridge JJs based on other superconducting materials. The spatial resolution of electron beam resists is sufficient for the preparation of nanobridges, in which all three dimensions (length, thickness and width) are equal to or smaller than the superconducting coherence length in this material, thereby fulfilling the conditions for “ideal” JJs that were formulated by K. K. Likharev [1]. Moreover, by using an undercut effect during RIE, it is possible to realize variable thickness nanobridge JJs, whose thickness is much smaller in the middle of the nanobridge when compared to the thickness of the same film in the nearby area of the electrodes. This is an important boundary condition for the theoretical description of nanobridge JJs [19,20,21]. Thick superconducting electrodes are also a precondition for low kinetic inductance of the SQUID loop, which is important for relatively large critical current modulation *I_c_*(*B*) and high resolution of the SQUIDs.

The much higher critical current density in the nanobridge JJs is associated with the different physical nature of the transport of Cooper pairs, when compared to the dominant contribution of the tunneling current-transport mechanism in the supercurrent in superconductor-insulator-superconductor (SIS) tunnel JJs (see [1], [22], etc.). The Ginzburg–Landau (GL) equation, which is appropriate for nanobridge JJs, can be written in a one-dimensional approximation in the form ([22,23,24])

(1) $$\xi^{2}\frac{d^{2}f}{dx^{2}}+f-f^{3}=0$$

where *ξ* is the coherence length and f=ΨΨ∞ is the normalized order parameter. In the case of a short length of nanobridge *L*<<ξ, the first term dominates and the equation is reduced to the Laplace equation *d*^2^*f*/*dx*^2^ = 0, for which the solution is the linear function *f = a + bx*, with *x* ranging from 0 to *L*

(2) $$f=\left(1-\frac{x}{L}\right)+\left(\frac{x}{L}\right)e^{i\Delta \varphi }$$

where Δφ is the phase difference between the wave functions in the electrodes. Insertion of the latter equation for *f* into the GL equation for the superconducting current *I_s_* results in the Josephson current-phase dependence *I_s_ = I_c_*sin(Δφ).

The energy sensitivity of the nanoSQUID at the operating temperature of 4.2 K is limited to a first approximation by Johnson noise, according to the expression [25].

(3) SΦ2L=SV(∂V∂Φ)22Lk=16kBTRN(∂V∂Φ)22Lk≅12 ℏ

where the values *R_N_* = 0.08 Ω and *∂V⁄∂*Φ ≅ 126 µV/Φ_0_ were measured from Figure 9. The relatively low intrinsic energy noise of ~12 ħ results from the very low resistance *R_N_*. The kinetic inductance of a nanoSQUID loop with variable-thickness nanobridge JJs *L_k_* is approximately 8 pH. We estimate the nanoSQUID to have a flux sensitivity *S*_Φ_^1/2^ ≅ 68 nΦ_0_/Hz, a magnetic field resolution *B_n_* = (∂*B*⁄∂Φ) *S*_Φ_^1/2^ ≅ 0.4 nT/√Hz and a spin sensitivity *S_n_*^1/2^ = *S*_Φ_^1/2^*r*/*r_e_* ≅ 11 μ_B_/√Hz, where *r_e_* = 2.82 × 10^−15^ m is the classical electron radius [26].

The influence of quantum fluctuations on the operation of TiN nanoSQUIDs at 4.2 K can be neglected. However, it cannot be excluded in the case of their potential applications at temperatures below 100 mK. The large coherence length *ξ*(0) of the TiN films allows the realization of non-hysteretic variable-thickness TiN nanobridge JJs in the milliKelvin temperature range, as well as their combination with high quality resonators made from the same material.

## 5. Conclusions

The observed large coherence length *ξ*(4.2 K) ~ 105 nm in TiN thin films allows relatively easy patterning of variable thickness nanobridge JJs with length ~100 nm, *J_c_* ~ 10 MA/cm^2^ and non-hysteretic *I*(*V*) characteristics at 4.2 K. NanoSQUIDs based on variable thickness nanobridge JJs demonstrate relatively low kinetic inductance of the loops and up to ~17 µV peak-to-peak modulation of the *V*(*B*) characteristics at 4.2 K. The high corrosion resistance of TiN provides long term stability of nanoSQUIDs and other superconducting circuits that are based on TiN nanobridge JJs. The technology offers prospects for superconducting nanobridge electronics and quantum computing.
